# Relationship between early use of tocilizumab during chimeric antigen receptor T-cell therapy for multiple myeloma and cardiovascular risk and progression-free survival

**DOI:** 10.1093/ehjopen/oeag097

**Published:** 2026-06-09

**Authors:** Sophia Golec, Weijia Fu, Erin Moshier, Ariel Peleg, Brunna Pileggi, Adriana Rossi, Gagan Sahni

**Affiliations:** Tufts Medical Center, 800 Washington St, Boston, MA 02111, USA; Mount Sinai Hospital, 1 Gustave Levy Pl, New York, NY 10029, USA; Mount Sinai Hospital, 1 Gustave Levy Pl, New York, NY 10029, USA; Jusidman Cancer Center, Institute of Oncology, Derech Sheba 2, Sheba Medical Center, Ramat Gan 52621, Israel; Department of Cardio-Pneumonology, Heart Institute of the University of Sao Paulo, Av. Dr. Enéas Carvalho de Aguiar, 44, Sao Paulo, Sao Paulo 05403, Brazil; Center of Excellence for Multiple Myeloma in the Tisch Cancer Institute at Mount Sinai Hospital, 1 Gustave Levy Pl, NewYork, NY 10029, USA; Mount Sinai Hospital, 1 Gustave Levy Pl, New York, NY 10029, USA; Zena and Michael A. Wiener Cardiovascular Institute Icahn School of Medicine at Mount Sinai, 1 Gustave Levy Pl, New York, NY 10029, USA

## Abstract

**Background:**

Chimeric antigen receptor-T-cell (CAR-T) therapies are approved for refractory and relapsed multiple myeloma (MM). Patients with MM have high cardiovascular disease burden due to age and cardiotoxic treatments. Retrospective studies in patients who develop severe grades of cytokine release syndrome (CRS) demonstrated a 20–30% major cardiovascular adverse event (MACE) rate. Early use of an interleukin-6 receptor antagonist, tocilizumab, to reverse CRS may lower rates of MACE.

**Aims:**

This observational study sought to examine the relationship between early use of tocilizumab and the development of MACE.

**Methods:**

We performed a retrospective chart review of adults undergoing CAR-T therapy for MM between 2017 and 2023 at a single medical center. Major cardiovascular adverse event was defined as a composite of myocardial injury, heart failure, stroke, arrhythmias, and cardiovascular death over a median period of 12 months. The secondary outcomes were progression-free survival (PFS) and overall survival (OS). Major cardiovascular adverse event incidence was estimated using the cumulative incidence function with non-cardiovascular death as a competing risk and compared by Gray’s test, and PFS/OS were analysed using Kaplan–Meier methods with log-rank tests.

**Results:**

Of the 145 patients studied, CRS occurred in 120 patients (82%). Of those with CRS, 107 (89%) received tocilizumab within 24 h. Within 1 year of therapy, 14 patients (9.7%) experienced MACE. Only patients with CRS developed MACE. The incidence of MACE was 8% [95% confidence interval (CI): 5%, 14%] at 6 months and 11% (95% CI: 7%, 19%) at 12 months. Progression-free survival at 12 months was 58% (95% CI: 50%, 68%).

**Conclusions:**

Progression-free survival and OS did not differ significantly based on incidence on CRS.

## Introduction

Chimeric antigen receptor-T-cell (CAR-T) therapy is a rapidly evolving therapy for relapsed and refractory haematologic malignancies. Its efficacy was initially established for a variety of leukaemia and lymphomas, including diffuse large B-cell lymphoma and mantle cell lymphoma.^1–4^ Cytokine release syndrome (CRS) is a common side effect of CAR-T-cell therapy associated with the activation and proliferation of T cells, characterized as a systemic inflammatory response.^5^ Patients with more severe CRS have been found to have worse overall outcomes compared with those who do not, including more major adverse cardiovascular events (MACEs).^6,7^ As CAR-T therapy becomes more common, the study of the prevention and management of CRS is of great clinical relevance.

Chimeric antigen receptor-T-cell therapy has been approved for the indication of relapsed and refractory multiple myeloma (MM).^8,9^ The indications continue to expand, now including those with lenalidomide-refractory disease and specifically triple-class exposed relapsed and refractory myeloma.^10,11^ This patient population is at high baseline risk of cardiovascular (CV) disease, both because patients present at an advanced age with pre-existing CV risk factors and because prior treatment with anthracyclines, proteasome inhibitors, stem cell transplants, and mediastinal radiation therapy are associated with adverse CV outcomes.^12,13^ Preventing additional MACE secondary to CRS in this patient population is likely of therapeutic importance.

Current practice guidelines have no recommendations on the timing of tocilizumab use during CRS due to a paucity of data.^14^ Tocilizumab, an interleukin-6 receptor antagonist, is an immunomodulatory agent that successfully attenuates the severity of CRS.^15^ There was a theoretical concern that tocilizumab could impact the anti-neoplastic treatment effect of CAR-T therapy, leading to initial reservation of its use to more severe cases of CRS.^16^ However, subsequent studies demonstrate that it does not affect progression-free survival (PFS) in B-cell acute lymphoblastic leukaemia.^17^ Tocilizumab was administered in 88 of the patients who experienced CRS during the initial CARTITUDE-1 trial, and it did not impact efficacy.^9^

There is an increasing body of evidence that early treatment of CRS attenuates MACE, including a recent prospective study.^18^ The aim of this study is to examine the relationship between early administration of tocilizumab of CRS among patients with relapsed or refractory MM and the development of MACE as well as PFS in patients with high baseline CV risk.

## Methods

We conducted a retrospective cohort analysis of all patients who received CAR-T therapy for the indication of MM at our single urban academic medical centre, The Mount Sinai Hospital in New York, NY. Patients’ electronic health records (EHRs) were manually reviewed from 2017 until June 2023. Major adverse cardiovascular events were confirmed by an additional physician. All research was approved by the Institutional Review Board. This was an observational cohort study, where data was collected using detailed retrospective EHR review. No written consent was required given the nature of the study. Inclusion criteria were patients who received CAR-T therapy for the indication of MM and who also had a baseline transthoracic echocardiogram prior to initiation of CAR-T therapy as a comparator to assess for potential left ventricular ejection fraction (LVEF) decline. Exclusion criteria included no echocardiogram data prior to therapy.

### Covariates

Covariates extracted from the EHR included sex assigned at birth and age. Cardiovascular risk factors included prior diagnosis of hypertension, hyperlipidaemia, diabetes mellitus, any former smoking, coronary artery disease, cerebrovascular accident, heart failure, hospitalization for heart failure, atrial fibrillation (AF), or other tachycardia. Other tachycardia was any other cause of tachycardia besides sinus tachycardia, including atrial flutter, atrial tachycardia, and ventricular tachycardia. Treatment with aspirin, angiotensin-converting enzyme inhibitor or angiotensin receptor blocker, beta-blocker, statin, oral hypoglycaemic agent, or insulin prior to CAR-T therapy was noted. Baseline CV data were described as the most recent troponin-I and brain natriuretic peptide (BNP) value prior to therapy, date of echocardiogram, and LVEF. Levels of haemoglobin, white blood cell count, platelet count, creatinine, glomerular filtration rate, and systolic and diastolic blood pressures were collected at the time of infusion of CAR-T cells. Oncologic history collected included any lifetime prior exposure to an anthracycline, exposure to carfilzomib within 1 year of CAR-T infusion, history of stem cell transplant, and history of mediastinal radiation. Mediastinal radiation was defined as any radiation from the clavicle to the 12th rib. The date of CAR-T infusion and product administered were collected. Presence or absence of CRS was documented as in the oncologist’s progress notes, which was scored according to the guidelines published by the American Society for Transplantation and Cellular Therapy consensus grading.^19^ If CRS occurred, further data was collected, including days to CRS from CAR-T infusion, days to administration of tocilizumab, and CRS grade.

### Primary and secondary outcomes

The primary outcome was the composite of MACEs. The composite score included myocardial injury, cerebrovascular accident, tachyarrhythmia, heart failure, or CV death following CAR-T therapy. Myocardial injury was defined as an increase in troponin-I above the upper limit of normal (>0.03 ng/dL). Cerebrovascular accident was defined as any new ischaemic or haemorrhagic cerebrovascular event as well as any diagnosed transient ischaemic accident. Atrial fibrillation was defined as an electrocardiogram with *de novo* AF. Other tachyarrhythmia included a search for atrial flutter, atrial tachycardia, or ventricular tachycardia. Heart failure was defined as a decrease in LVEF by 10% or a decrease to below 55% or a new diagnosis of clinical heart failure in the electronic medical record. Cardiovascular death was defined by a discharge diagnosis of death due to a primarily CV cause, as collected from the Mount Sinai EHR.

The secondary outcomes were PFS and overall survival (OS) from MM following treatment with CAR-T therapy. Progression of disease was defined according to the International Myeloma Working Group consensus criteria for response and minimal residual disease in MM.^20^ Overall survival was determined via chart review of all patients up until the completion of the study time frame.

### Statistical analysis

Baseline characteristics were summarized for the total cohort and stratified by the occurrence of CRS during follow-up. Categorical variables were reported as counts and percentages, and continuous variables as medians with interquartile ranges (IQRs). Group comparisons were performed using the χ^2^ test or Fisher’s exact test for categorical variables and the Wilcoxon rank-sum test for continuous variables, as appropriate.

Time to MACE was defined as the time from CAR-T cell infusion to the first documented MACE. Patients without a MACE were censored at the date of the last follow-up. The cumulative incidence of MACE was estimated using the cumulative incidence function (CIF), with non–cardiac-related death treated as a competing risk, and groups were compared using Gray’s test. Cumulative incidence function point estimates with 95% confidence intervals (CIs) were reported at 6 and 12 months.

Progression-free survival was defined as the time from CAR-T cell infusion to documented disease progression or death from any cause, whichever occurred first. Patients without progression or death were censored at the date of the last follow-up. Overall survival was defined from infusion to death from any cause; survivors were censored at the last follow-up. Progression-free survival and OS were estimated using the Kaplan–Meier method, and survival curves were compared between CRS and no CRS using the log-rank test. Kaplan–Meier survival probabilities at select time points with 95% CIs were calculated using Greenwood’s variance with the log–log transformation, and median PFS was reported with Brookmeyer–Crowley 95% CIs. Median follow-up time was estimated using the reverse Kaplan–Meier method.

All statistical tests were two-sided, and *P* < 0.05 were considered statistically significant. Analyses were conducted using SAS version 9.4 (SAS Institute, Cary, NC).

## Results

### Baseline characteristics

A total of 145 patients met the inclusion criteria. The baseline characteristics of the total cohort are summarized in *Table 1*. The median age was 61 years at the time of infusion (IQR: 56–68), and 88 (61%) were male. Of all patients studied, 45 patients (31%) had hypertension, 53 (37%) were prior or current smokers, 51 (35%) had hyperlipidaemia, 33 (23%) had diabetes mellitus, 24 (17%) had coronary artery disease, 20 (14%) had heart failure, 3 (2%) had a prior hospitalization secondary to heart failure exacerbation, 13 (9%) had AF, 8 (6%) had a history of cerebrovascular accident (CVA), and 4 (3%) had an additional tachyarrhythmia. Among patients with heart failure, eight (6%) had an ejection fraction of <50% on their baseline echocardiogram. There was mostly no statistically significant difference between patients who did and did not develop CRS. However, patients who developed CRS were older (*P* < 0.05) and were less likely to have had prior mediastinal radiation (*P* < 0.05). There were also no significant differences between baseline characteristics of patients who developed MACE (*n* = 14) and the remainder of the cohort (see Supplementary material online, *Table S1*).

**Table 1:** Baseline characteristics

				
	Total Cohort (*n* = 145)	CRS (*n* = 120)	No CRS (*n* = 25)	*P* value
Male	88 (61%)	72 (60%)	16 (64%)	0.7095
Age at infusion, median [Q1, Q3]	61.0 [56.0, 68.0]	63.0 [57.0, 70.0]	59.0 [55.0, 64.0]	0.0181
Past medical history				
Hypertension	45 (31%)	41 (34%)	4 (16%)	0.0966
Smoking history	53 (37%)	44 (37%)	9 (36%)	0.9498
Hyperlipidaemia	51 (35%)	43 (36%)	8 (32%)	0.7150
DM	33 (23%)	26 (22%)	7 (28%)	0.4920
CAD	24 (17%)	21 (18%)	3 (12%)	0.7674
Heart failure	20 (14%)	17 (14%)	3 (12%)	1.0000
Atrial fibrillation	13 (9%)	9 (8%)	4 (16%)	0.2397
CVA	8 (6%)	8 (7%)	0 (0%)	0.3513
Tachyarrhythmia	4 (3%)	3 (3%)	1 (4%)	0.5350
HF hospitalization	3 (2%)	3 (3%)	0 (0%)	1.0000
Medications				
Aspirin	59 (41%)	51 (43%)	8 (32%)	0.3309
Statin	37 (26%)	29 (24%)	8 (32%)	0.4138
ACE-i/ARB	36 (25%)	33 (28%)	3 (12%)	0.1297
Beta-blocker	36 (25%)	28 (23%)	8 (32%)	0.3615
Oral hypoglycaemic	12 (8%)	10 (8%)	2 (8%)	1.0000
Injectable insulin	2 (1%)	1 (1%)	1 (4%)	0.3161
Oncologic history				
Prior stem cell transplant	117 (81%)	99 (83%)	18 (72%)	0.2263
Carfilzomib within 1 year	68 (47%)	59 (49%)	9 (36%)	0.2301
Prior anthracycline treatment	36 (25%)	30 (25%)	6 (24%)	0.9161
Prior mediastinal radiation	35 (24%)	25 (21%)	10 (40%)	0.0442
Blood pressure, median [Q1, Q3]				
Systolic blood pressure (mmHg)	116.0 [107.0, 127.0]	115.5 [106.0, 126.0]	119.0 [110.0, 128.0]	0.3876
Diastolic blood pressure (mmHg)	66.0 [59.0, 72.0]	65.5 [58.5, 71.0]	66.0 [60.0, 76.0]	0.1851
LVEF, median [Q1, Q3]	60.0 [58.0, 65.0]	60.0 [58.0, 65.0]	60.0 [60.0, 65.0]	0.2152
Laboratory data, median [Q1, Q3]				
Haemoglobin (g/dL)	9.6 [8.4, 10.6]	9.6 [8.4, 10.6]	9.6 [8.5, 11.1]	0.5027
White blood cell count (1000/uL)	1.5 [0.9, 2.3]	1.5 [0.9, 2.3]	1.8 [1.0, 2.3]	0.4633
Platelet count (1000/uL)	146.0 [108.0, 190.0]	147.5 [109.5, 190.5]	142.0 [105.0, 188.0]	0.6043
Creatinine (mg/dL)	0.8 [0.7, 0.9]	0.8 [0.7, 0.9]	0.8 [0.6, 0.9]	0.3473
CAR-T product				
Abecma	60 (41%)	48 (40%)	12 (48%)	0.7024
Carvykti	67 (46%)	55 (46%)	12 (48%)	
Research product	18 (12%)	17 (14%)	1 (4%)	

Baseline characteristics for the total cohort (*n* = 145) as well as those characteristics for patients who developed CRS (120) and those who did not (25).

Patients’ most recent medication use at the time of treatment included 59 prescribed aspirin (41%), 37 prescribed any form of a statin (26%), 36 prescribed an angiotensin-converting enzyme inhibitor or angiotensin receptor blocker (25%), 36 prescribed a beta-blocker (25%), 12 prescribed an oral hypoglycaemic (8%), and 2 prescribed injectable insulin (1%).

Baseline objective data included an average ejection fraction of 60% (IQR: 58–65). At the time of infusion, systolic blood pressure was an average of 116 mmHg (IQR: 107–127) and a diastolic blood pressure of 66 mmHg (IQR: 59–72). The patient’s baseline laboratory values demonstrated an average haemoglobin of 9.6 g/dL (IQR: 8.4–10.6), a median white blood cell count of 1.5 k/uL (IQR: 0.9–2.3), and a platelet count of 146 K/uL (IQR: 108–190). The average creatinine was 0.8 mg/dL (IQR: 0.7–0.9).

Regarding oncologic history, there were 117 patients with prior stem cell transplants (81%), 68 patients with carfilzomib exposure within 1 year of CAR-T therapy (47%), 36 patients with prior anthracycline treatment (25%), and 35 with prior mediastinal radiation (24%). All patients had a prior diagnosis of MM given the inclusion criteria. For the CAR-T product administered, 67 (47%) received the commercial CAR-T carvykti (ciltacabtagene autoleucel; Janssen Biotech, Horsham Township, PA), 60 (41%) received Abecma (idecabtagene vicleucel; Bristol-Myers Squibb Company, Lawrence, NJ), and the remaining 18 (2%) received an investigational product. Those CAR-T products included Caribou Biosciences, Sorrento Therapeutics, and Allogene Therapeutics.

### Cytokine release syndrome incidence and tocilizumab administration

A total of 120 patients (82% of all patients) had the side effect of CRS (*Table 2*). Of those, 98 patients had a maximum of Grade 1 (68%), 19 had Grade 2 (13%), and 3 had Grade 3 (2%). Of the 120 patients who experienced CRS, 107 received tocilizumab (89%). The days to development of CRS after CAR-T administration were an average of 6 days (CI: 4–7). Most patients received tocilizumab within the first day of the onset of CRS. Most patients who did not receive tocilizumab only experienced Grade 1 toxicity.

**Table 2:** Cytokine release syndrome and treatment with tocilizumab

	Total cohort (*n* = 145)
CRS grade, *n* (%)	
No CRS	25 (17%)
Grade 1	98 (68%)
Grade 2	19 (13%)
Grade 3	3 (2%)
Days to CRS after CAR-T, median [95% CI]	6.0 [4.0, 7.0]
Days to Toci after CRS, median [95% CI]	0.0 [0.0, 1.0]
Patients given tocilizumab by CRS grade, *n* (% per grade of CRS)	
Grade 1	86 (88%)
Grade 2	18 (95%)
Grade 3	3 (100%)

The number of patients who developed cytokine release syndrome and the percentage of each grade of CRS from 1 to 3. Also described is the median age to CRS after CAR-T treatment as well as the median days to administration of tocilizumab from CRS development. Among those with CRS, the number and percentage of patients who received tocilizumab by grade of CRS is listed.

### Cardiovascular events following chimeric antigen receptor-T-cell therapy

**Figure 1:**
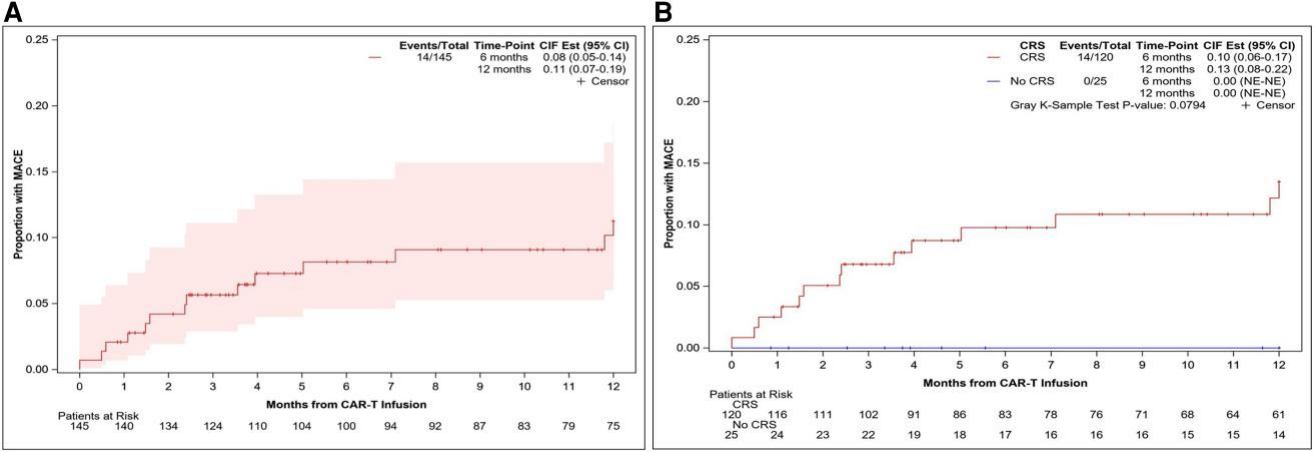
Patients were followed for development of major adverse cardiovascular events. (*A*) The cumulative incidence function curve of the time to development of major adverse cardiovascular events after treatment with chimeric antigen receptor-T-cell therapy. (*B*) No patients in the no cytokine release syndrome cohort developed major adverse cardiovascular events. There were 21 instances of major adverse cardiovascular events across the 14 patients who developed major adverse cardiovascular events during the study. The subtypes of major adverse cardiovascular events that the patients developed are listed here.

There was a total of 14 MACEs within 1 year of CAR-T therapy for a total incidence of 9.7% among the patients studied (*Figure 1A*). The rate of MACE within the first 6 months was 8% (CI: 5–14%) and within 12 months was 11% (CI: 7–19%). Among the 14 patients who developed MACE, there were 21 individual adverse events. There were seven patients who developed new heart failure (33% of events), five patients with myocardial injury (24%), two patients with new stroke (10%), three patients with AF (14%), and three patients with other tachyarrhythmias (14%) (*Figure 1*). Among the five patients with myocardial injury, no patients required urgent revascularization, although one patient ultimately expired due to cardiac arrest. Among the seven patients who developed new heart failure, five patients had a reduction in LVEF to <50%, one patient had a reduction from 65 to 50%, and one patient had an exacerbation of heart failure with preserved ejection fraction. No MACEs occurred among patients who did not develop CRS, although the difference in MACE between patients who did and did not develop CRS did not reach statistical significance (*P* = 0.08) (*Figure 1B*).

**Table:** 

	Total cohort	CRS	No CRS	*P* value
	(*n* = 145)	(*n* = 120)	(*n* = 25)	
**Major adverse cardiovascular events (MACEs)**				0.0794
MACE, *n* (%)	14 (9.7%)	14 (11.7%)	0 (0%)	
Deaths, *n* (%)				
Alive without MACE, *n* (%)				
6-month MACE, CIF [95% CI]	8% [5%, 14%]	10% [6%, 17%]	0%	
12-month MACE, CIF [95% CI]	11% [7%, 19%]	13% [8%, 22%]	0%	
# MACE events	21	21	0	
Heart failure, *n* (%)	7 (33%)	7 (33%)	**—**	
Myocardial injury, *n* (%)	5 (24%)	5 (24%)	**—**	
Cardiovascular death, *n* (%)	1 (5%)	1 (5%)	**—**	
CVA, *n* (%)	2 (10%)	2 (10%)	**—**	
Atrial fibrillation, *n* (%)	3 (14%)	3 (14%)	**—**	
Other tachyarrhythmia, *n* (%)	3 (14%)	3 (14%)	**—**	
*P* value from Gray’s test (CRS vs. no CRS). Percentages use column denominators. There were 21 instances of MACE across the 14 patients who developed MACE during the study. The subtypes of MACE that the patients developed are listed here. CIF, cumulative incidence function.				

### Progression-free survival and overall survival

**Figure 2:**
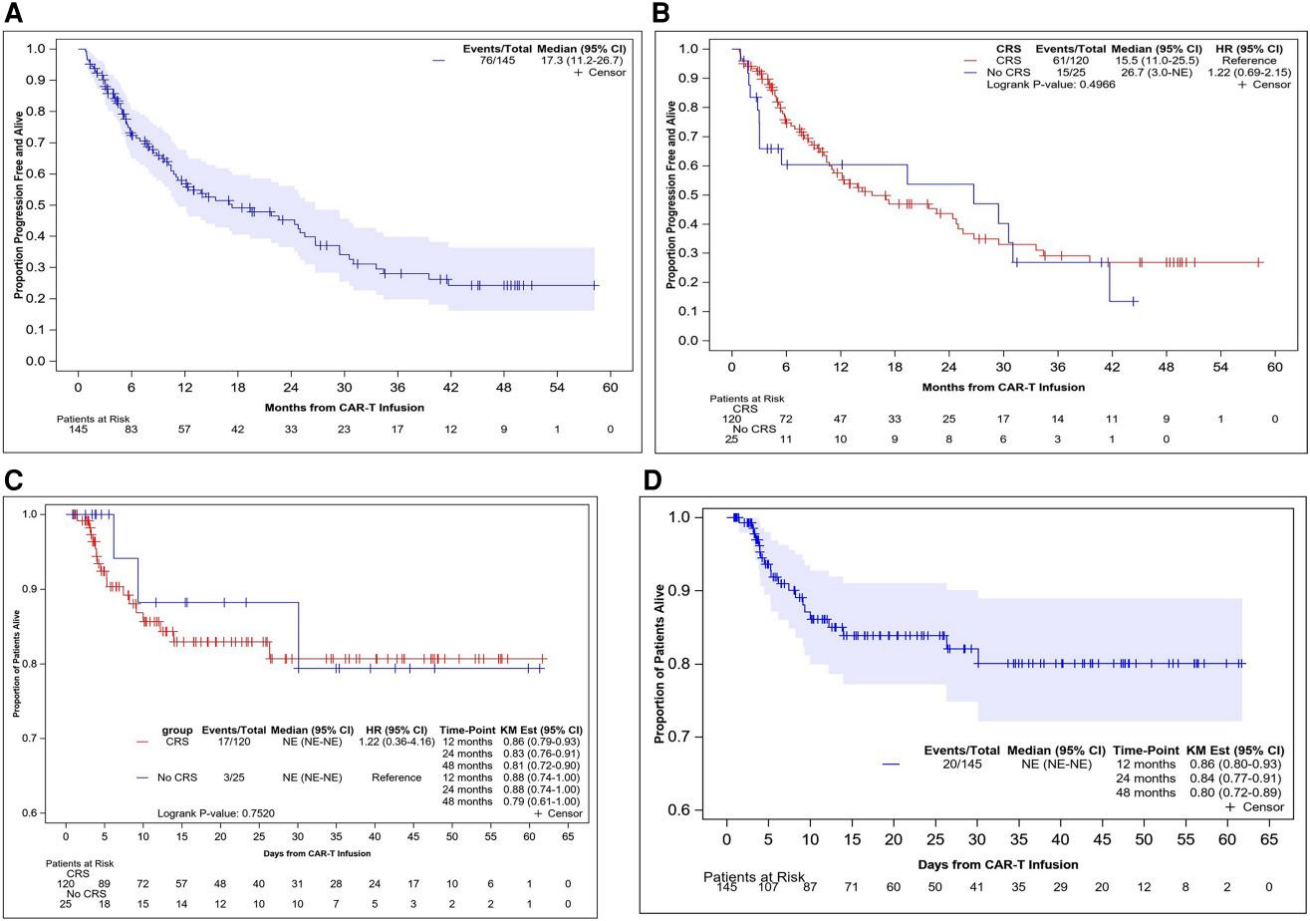
Patients were followed until progression of disease or death. (*A*) The Kaplan–Meier curve of progression-free survival among all patients (*B*) and differentiated by those who developed cytokine release syndrome or not. (*C*) The Kaplan–Meier curve of the overall survival among all patients and (*D*) among patients who developed cytokine release syndrome and those who did not.

Within the total cohort, 67 patients (46%) developed progression of disease, 9 patients (6.2%) died, and 67 (46.2%) were censored (*Figure 2A* and *B*). The 6-month rate of PFS as per Kaplan–Meier survival curve was 72% (CI: 65–81%), at 12 months it was 58% (CI: 50–68%), at 24 months it was 45% (CI: 36–56%), and at 48 months it was 24% (CI: 16–36%). There were no significant differences between the rate of progression of disease among patients who developed CRS vs. those who did not develop CRS (*P* = 0.49) (*Figure 2A* and *B*). At the end of the study period, 20 patients (13%) had died, of which 17 (14.2%) had developed CRS and 3 (12%) had not (*P* = 0.7) (*Figure 2C* and *D*). The median follow-up time was 19.5 months (IQR: 15.2–25.5) for OS and 27.3 months (IQR: 18.4–41.5) for PFS.

**Table:** 

	Total cohort	CRS	No CRS	*P* value
	(*n* = 145)	(*n* = 120)	(*n* = 25)	
**Progression-free survival (PFS)**				0.4966
Progressions, *n* (%)	67 (46.2%)	53 (44.2%)	14 (56.0%)	
Deaths, *n* (%)	9 (6.2%)	8 (6.7%)	1 (4.0%)	
Alive without progression, *n* (%)	69 (47.6%)	59 (49.2%)	10 (40.0%)	
Median PFS, months [95% CI]	17.3 [11.2, 26.7]	15.5 [11.0, 25.5]	26.7 [3.0, NE]	
6-month PFS, KM [95% CI]	0.72 [0.65, 0.81]	0.75 [0.67, 0.84]	0.60 [0.43, 0.85]	
12-month PFS, KM [95% CI]	0.58 [0.50, 0.68]	0.58 [0.48, 0.68]	0.60 [0.43, 0.85]	
24-month PFS, KM [95% CI]	0.45 [0.36, 0.56]	0.44 [0.34, 0.56]	0.54 [0.36, 0.81]	
**Overall survival (OS)**				0.7520
Deaths, *n* (%)	20 (13.8%)	17 (14.2%)	3 (12.0%)	
Alive, *n* (%)	125 (86.2%)	103 (85.8%)	22 (88.0%)	
12-month OS [95% CI]	0.86 [0.80, 0.93]	0.86 [0.79, 0.93]	0.88 [0.74, 1.00]	
24-month OS [95% CI]	0.84 [0.77, 0.91]	0.83 [0.76, 0.91]	0.88 [0.74, 1.00]	
48-month OS [95% CI]	0.80 [0.72, 0.89]	0.81 [0.72, 0.90]	0.79 [0.61, 1.00]	
*P* values from log-rank tests (CRS vs. no- CRS). Percentages use column denominators. KM, Kaplan–Meier estimate; NE, not estimable.				

## Discussion

In this observational retrospective cohort study of patients with refractory and relapsed MM treated with CAR-T, MACE remained low when compared with previous studies while PFS was preserved.

Our study cohort had a high baseline CV risk, with a median age of 61 and a significant portion with a history of smoking, hypertension, and diabetes. They also had a relatively high incidence of pre-existing CV disease, including coronary artery disease, hyperlipidaemia, diabetes, CVA, heart failure, and tachyarrhythmia. The cohort was reasonably diverse, with 39% women. Additionally, since this was a study of only patients with relapsed or refractory MM, a large portion of the cohort had previous exposure to multiple lines of potentially cardiotoxic therapies for MM, including anthracyclines, carfilzomib within 1 year of CAR-T therapy, mediastinal radiation, and stem cell transplants.

While most patients developed some form of CRS (82%), most patients had only Grade 1 CRS (68%), which was likely due to early use of tocilizumab in the majority of the patients within 1 day of CRS. Those that developed CRS were likely to be older with a history of HTN, and the cohort without CRS were younger patients with a prior history of mediastinal radiation (*Table 1*). Despite the high burden of CV disease among our patient population, the incidence of MACE, which included myocardial injury, cerebrovascular accident, AF or other tachyarrhythmia, heart failure, or cardiovascular death, occurred in only 14 of 145 patients (9.7%) at 1 year. This rate is significantly lower than what was demonstrated in previous studies with shorter follow-up.^6,21–23^ Moreover, there was no difference in PFS or OS in patients who developed CRS and received tocilizumab vs. those who did not develop CRS. Given the low sample size, our analysis was unable to directly compare CV outcomes between patients who developed CRS who did and did not receive tocilizumab, limiting interpretation of these results.

In this single-centre, retrospective observational cohort study, we observed that early administration of tocilizumab was associated with a relatively low incidence of MACEs. Most previous studies with higher rates of MACE after CRS were early studies of CAR-T outcomes where levels of CRS grade were significantly higher.^21^ More recent prospective data support the relatively low rate of cardiotoxicity of CAR-T.^18^ This decrease in MACE likely correlates with changes in treatment patterns to emphasize preventing higher grades of CRS.

Importantly, our patients also had a rate of 56% PFS over a 1-year follow-up period and 43% over a 24-month period. This is in contrast to the original PFS as demonstrated in the CARTITUDE trials, which were 77% within 1 year and 54.9% at 27 months.^9,24^ This is likely because of the difference between patients selected for inclusion in clinical trials vs. patients in the real-world setting. Among patients who did develop CRS and therefore mostly received tocilizumab, the PFS did not differ significantly. While the lack of a control group who did not receive tocilizumab limits the interpretation of our study, the preservation of anti-neoplastic efficacy after tocilizumab administration is supported by previous studies. Banerjee *et al.*^25^ showed that patients who received early tocilizumab (<12 h from the onset of CRS symptoms) do not appear to compromise efficacy when compared with patients who received the medication later (Day +30 objective response 74% vs. 79%, with early and late administration, respectively; *P* = 0.03). Costa *et al.*^26^ published additional retrospective data confirming that PFS is preserved in MM patients treated with tocilizumab or steroids for CRS or Immune Effector Cell-Associated Neurotoxocity Syndrome.

The use of prophylactic tocilizumab is already under active research. One study investigated the prophylactic use of tocilizumab for MM patients treated with the bispecific antibody targeting B-cell maturating antigen teclistimab.^27^ Patients were less likely to develop CRS, and PFS was not adversely affected. Prophylactic tocilizumab was studied in a small group of patients treated with anti-CD19 CAR-T cells for non-Hodgkin lymphoma with lower incidence of CRS and no adverse events or effect on disease control.^28^ Alternatively, Locke *et al.*^29^ studied the prophylactic use of tocilizumab in patients treated with CAR-T therapy for relapsed or refractory large B-cell lymphoma and did not see a decrease in CRS grade. However, there was concern that tocilizumab masked early presentation of CRS and could have led to a higher cytokine concentration leading to higher grade CRS. The use of prophylactic tocilizumab has not been studied in patients with relapsed and refractory MM treated with CAR-T therapy. Further studies are needed to determine the potential benefit of pre-emptive use to prevent MACE, reduce intensive care time and overall length of stay, use of vasopressors, etc., particularly in myeloma patients who have a background of higher CV risk. Alternative treatment modalities for the prevention of CRS are also under active research, including the interleukin-1 receptor antagonist anakinra and the tumour necrosis factor-alpha blocking antibody etanercept, although neither has been approved for routine use.^30,31^ The pre-emptive use of these drugs also warrants being studied for their role in preventing neurotoxicity during CAR-T.

To our knowledge, there is no previously published study that describes both the CV outcomes and the rate of PFS following early tocilizumab administration in patients treated with CAR-T for relapsed or refractory MM over a multi-year observation period.

This study has several key limitations. This single-centre, retrospective, observational cohort study included a robust patient population of 145 patients. However, the majority of the patients developed CRS and thus received tocilizumab, leading to a lack of a true comparator group of patients with CRS who did not receive tocilizumab. This limits the ability to compare if tocilizumab leads to clinically significant differences in MACE or PFS. Our real-world observational data of the low incidence of MACE among patients with elevated baseline CV risk treated with CAR-T therapy is in keeping with increasing evidence for the relative CV safety of CAR-T therapy. However, our low incidence of MACE events (*n* = 14) further limits the power of the study. Since this was a retrospective study, the decision for tocilizumab administration and timing were according to clinician discretion and not standardized, though all doses were within 24 h of CRS. Therefore, some patients who developed CRS did not receive tocilizumab. In addition, cardiac biomarker and imaging were not obtained in a standardized fashion but rather as clinically indicated. Our study also did not analyse the role of early tocilizumab in preventing potential neurotoxicity from CAR-T. Assessment of MACE in our study included troponin elevation as a marker of myocardial injury, which did not necessarily indicate clinically significant MI. Due to the relatively recent approval of CAR-T therapy for MM, this multi-year cohort remained small and did not have the power to permit propensity score matching of outcomes among patients who developed cardiotoxicity and those who did not.

## Conclusions

Chimeric antigen receptor-T-cell therapy has transformed the treatment of haematological malignancies due to its efficacy and safety profile. Despite the limited number of studies evaluating CV outcomes among recipients of CAR-T cell therapy in MM, it appears to have a relatively safe CV profile especially with early use of tocilizumab. This real-world, observational cohort study did not find high incidence of MACE among patients with relapsed or refractory MM treated with early tocilizumab. Patients were also not observed to have rates of PFS that differ significantly from existing outcomes data among high-risk patients. Therefore, studies exploring the pre-emptive use of tocilizumab should be the focus of future CAR-T studies from both oncological and CV perspectives.

## Supplementary Material

oeag097_Supplementary_Data

## Data Availability

All data used in this study have been de-identified to remove any potential patient identifiers and comply with the Health Insurance Portability and Accountability Act in the United States. The de-identified dataset is available upon reasonable request.
