# Supplementary material for: Relationship between early use of tocilizumab during chimeric antigen receptor T-cell therapy for multiple myeloma and cardiovascular risk and progression-free survival
Source: Eur Heart J Open. 2026 Jun 9;6(3):oeag097. doi: 10.1093/ehjopen/oeag097 (PMC13308710; doi:10.1093/ehjopen/oeag097)
Supplement: oeag097_Supplementary_Data [file oeag097_supplementary_data.docx]

|  | **Total Cohort  (N = 145)** | **No MACE (N = 131)** | **MACE (N = 14)** | **P-value** |
| --- | --- | --- | --- | --- |
| **Male** | 88 (61%) | 79 (60%) | 9 (64%) | 1.0000 |
| **Past medical history** |  |  |  |  |
| Hypertension | 45 (31%) | 41 (31%) | 4 (29%) | 1.0000 |
| Smoking history | 53 (37%) | 50 (38%) | 3 (21%) | 0.2574 |
| Hyperlipidemia | 51 (35%) | 46 (35%) | 5 (36%) | 1.0000 |
| Diabetes mellitus | 33 (23%) | 26 (20%) | 7 (50%) | 0.0178 |
| Coronary artery disease | 24 (17%) | 22 (17%) | 2 (14%) | 1.0000 |
| Heart Failure | 20 (14%) | 17 (13%) | 3 (21%) | 0.4118 |
| Atrial Fibrillation | 13 (9%) | 12 (9%) | 1 (7%) | 1.0000 |
| Cerebrovascular accident | 8 (6%) | 6 (5%) | 2 (14%) | 0.1732 |
| Other tachyarrhythmia | 4 (3%) | 3 (2%) | 1 (7%) | 0.3368 |
| Heart failure hospitalization | 3 (2%) | 3 (2%) | 0 (0%) | 1.0000 |

Supplementary Table 1: Baseline characteristics for the total cohort (n=145) as compared between patients who developed MACE (n=14) and those who did not (n=131).
